# The effects of lutein/ zeaxanthin (Lute-gen^®^) on eye health, eye strain, sleep quality, and attention in high electronic screen users: a randomized, double-blind, placebo-controlled study

**DOI:** 10.3389/fnut.2025.1522302

**Published:** 2025-02-03

**Authors:** A. L. Lopresti, S. J. Smith

**Affiliations:** ^1^Clinical Research Australia, Perth, WA, Australia; ^2^Healthy Ageing Research Centre and Discipline of Psychology, College of Science, Health, Engineering and Education, Murdoch University, Perth, WA, Australia

**Keywords:** lutein, zeaxanthin, carotenoids, dry eyes, vision, electronic screen use, sleep

## Abstract

**Background:**

Lutein and zeaxanthin are fat-soluble antioxidant nutrients that have evidence of beneficial effects on vision and eye health.

**Purpose:**

Examine the effects of supplementation with lutein and zeaxanthin isomers (Lute-gen^®^) on eye health, eye strain, sleep quality, and attention in high electronic screen users.

**Study design:**

Two-arm, 6-month, parallel-group, randomized, double-blind, placebo-controlled trial.

**Methods:**

Seventy volunteers aged 18 to 65 who used electronic screens for more than 6 h daily were supplemented with 10 mg of lutein and 2 mg of zeaxanthin-isomers or a placebo. Outcome measures included several ophthalmic examinations comprising the Schirmer tear test, photo-stress recovery time, contrast sensitivity, tear film break-up time, and self-report measures of visual fatigue, computer vision, sleep quality and attention.

**Results:**

Compared to the placebo, lutein and zeaxanthin supplementation was associated with greater improvements in the Schirmer tear test, photo-stress recovery time, and tear film break-up time. However, there were no between-group differences in the change in self-report measures or contrast sensitivity. Lutein and zeaxanthin supplementation was well-tolerated, with no reports of serious adverse reactions or clinically significant changes in safety blood measures, including liver function, renal function, blood lipids, and full blood examination.

**Conclusion:**

The results from this study provide support for the beneficial effects of 6 months of lutein and zeaxanthin supplementation on regular users of electronic screens. Compared to the placebo, there were improvements in several ophthalmic examinations for dry eyes and visual health. However, these findings were not corroborated by group differences in the administered self-report measures. Lutein and zeaxanthin were well tolerated, with no serious adverse effects or significant changes in vital signs or blood safety measures.

## 1 Introduction

Eye and vision problems associated with high computer and electronic screen use are increasingly recognized as problems for the community. Symptoms can include ocular and visual symptoms such as itching, burning, dryness, blurred vision, and photophobia; and pain-related conditions such as headaches and neck and shoulder pain. Computer vision syndrome, visual fatigue, and digital eye strain are terms often used to reflect these symptoms (1). A commonly posited hypothesis for the relationship between digital screen use and ocular symptoms is that digital screen use changes blinking dynamics, leading to ocular dryness (2). Through overexposure to blue light, electronic screen use can also contribute to the excessive production and accumulation of free oxygen radicals in mitochondria and photosensitive molecules (3, 4).

Lutein and Zeaxanthin (LZ) are fat-soluble antioxidant nutrients in the carotenoid family. Lutein (L) is found in dark green leafy vegetables such as spinach and kale and in corn and egg yolks. Zeaxanthin (Z) is more prominent in orange and yellow foods such as corn, egg yolks, orange capsicums, persimmons, tangerines, mandarins, and oranges. In the body, LZ are found in the eye, brain, breast, and adipose tissue. Several studies have examined the effects of LZ on eye health, with mostly positive results (5). In several reviews and meta-analyses, it was concluded that LZ intake can help with ocular health and reduce the risk of some eye diseases (6–8). The effects of LZ on high-electronic screen users require further investigation; however, its six-month supplementation was associated with improvements in eye strain, eye fatigue, visual performance, sleep, and headache frequency (9). Moreover, another study found positive effects on dry eye symptoms after LZ supplementation in adults with dry eye syndrome (10). Physiological mechanisms that may account for the protective effects of LZ on vision may be through their antioxidant and anti-inflammatory effects (2, 5), and the ability of L to reduce phototoxic damage to photoreceptor cells after blue light exposure (11).

Given the preliminary positive evidence of LZ on eye health, this study aimed to investigate further the effects of LZ supplementation on high electronic screen users. It was hypothesized that LZ supplementation would positively affect ocular symptoms based on outcome measures comprising ophthalmic examinations and self-report questionnaires. The inclusion of subjective questionnaires and more objective ophthalmic examinations helped determine whether changes in objective parameters were associated with symptomatic changes that could be identified by participants. Moreover, as an exploratory investigation, the effects of LZ on sleep and attention were examined.

## 2 Materials and methods

### 2.1 Study design

**Figure 1:**
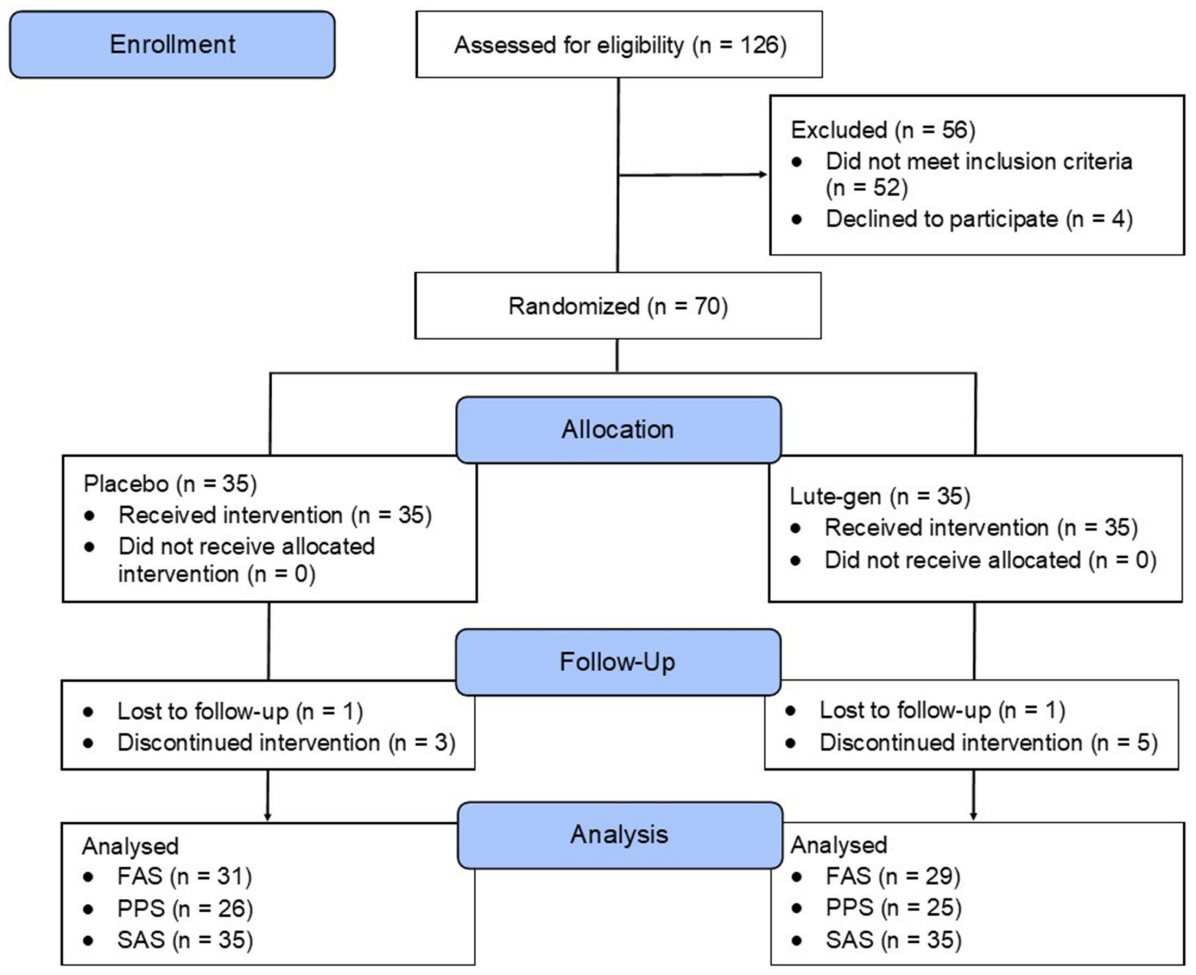
Systematic illustration of study design.

This was a 180-day (6 months), parallel-group, two-arm, randomized, double-blind, placebo-controlled trial (Figure 1). The study received ethics approval from the National Institute of Integrative Medicine Human Research Ethics Committee (approval number 0122E_2023), and informed consent was acquired from all participants. This trial was registered prospectively with the Australian and New Zealand Clinical Trials Registry (ACTRN12623000427673).

### 2.2 Recruitment and randomization

Between May 2023 and September 2023, social media advertisements and e-mail databases were used for volunteer recruitment. Eligible participants were randomly allocated to one of two groups (LZ or placebo; 1:1 ratio) using a randomization calculator with the randomization structure comprising 7 randomly permuted blocks, with 10 participants per block. A participant identification number was assigned based on the order of participant enrollment. The randomization sequence was generated by a researcher not directly involved in volunteer recruitment, and bottle codes were stored by the study sponsor and revealed after all data were analyzed. All softgels were packed in matching bottles. Researchers were blind to the treatment allocation until all outcomes were collected and a blind review was completed.

### 2.3 Participants

#### 2.3.1 Inclusion criteria

The inclusion criteria for the study comprised the following: Healthy adults (male and female) 18 to 65 years; devotes at least 6 h a day viewing a screen at a distance of 1 meter or less; non-smoker; body mass index (BMI) between 18 and 30 kg/m^2^; has no plan to commence new treatments over the study period; willing to maintain their current diet, exercise, and supplement regimen during the study period; and if wearing spectacles for vision, best corrected visual acuity must be 6/6.

#### 2.3.2 Exclusion criteria

The exclusion criteria for the study comprised the following: Ocular disorders including but not limited to cataracts, corneal diseases, ocular surface disorders, glaucoma, retinal disease, and myopia (except mild to moderate severity); undergone eye surgery in the past; wears contact lenses more than 3 days a week; suffering from a recently diagnosed or uncontrolled medical condition including but not limited to hyper/hypotension, diabetes, cardiovascular disease, gastrointestinal disease, gallbladder disease, rheumatoid arthritis or another autoimmune disease, endocrine disease, or cancer/malignancy; diagnosed with a psychiatric/neurological condition including but not limited to a severe psychiatric disorder (other than mild-to-moderate depression or anxiety); regular medication intake including but not limited to steroid medications, hormone replacement therapy, eye drops, antihistamines, beta-blockers, or tricyclic antidepressants; change in medication in the last 3 months or an expectation to change during the study duration; taking vitamins or herbal supplements that may affect the study measures; current or 12-month history of illicit drug use; alcohol intake more than 14 standard drinks per week; pregnant, breastfeeding, or an intention to fall pregnant in the next 6 months; any significant surgeries over the last year; and planned major lifestyle change in the next 6 months.

### 2.4 Interventions

The intervention comprised either a combination of lutein & zeaxanthin isomers (Lute-gen^®^) or a placebo (sunflower oil). Participants were required to take one capsule daily with a meal, with the active intervention delivering 10 mg of lutein and 2 mg of zeaxanthin-isomers daily for 180 days. The active and placebo soft gel capsules were identical in appearance, matched for shape, color, size, smell, and taste. The excipients in the soft gels were also identical, comprising sunflower oil. Adherence to intake was assessed by asking participants to estimate capsule intake consistency (0 to 100%) every month and by the return of unused capsules at the day 90 and 180 visits. Treatment blinding was assessed by asking participants to predict group allocation (placebo, lutein/zeaxanthin, or unsure) at the end of the study.

### 2.5 Outcome measures

#### 2.5.1 Primary outcome measures

##### 2.5.1.1 Visual fatigue scale (VFS)

The VFS is a 10-item questionnaire assessing symptoms of eye discomfort experienced after a typical workday. Symptoms are rated from 0 (none) to 6 (severe), with higher scores indicating greater visual fatigue (12).

##### 2.5.1.2 Schirmer tear test (STT)

The STT assesses tear production, especially in patients with suspected dry eye or tear overproduction. In the test, a special paper strip is placed inside the lower eyelid of each eye and bent at 90 degrees. The eyes are then closed for 5 min, after which time the paper is removed. The Schirmer test score is calculated by the length of the moistened area of the strips (using the scale included on the strips) and the measurement duration in minutes. A score of greater than 10 mm in 5 min is considered normal. A score of less than 5 mm in 5 min indicates a tear deficiency (13). A mean score for tests conducted on the left and right eye at each visit was calculated to evaluate changes over time.

### 2.6 Secondary outcome measures

#### 2.6.1 Computer vision syndrome questionnaire (CVS-Q)

The CVS-Q is a 16-item self-report measure assessing computer-related visual and ocular symptoms associated with computer/ screen use. Symptoms are rated based on frequency (never, occasional, often/always) and intensity (moderate and intense), with higher scores indicating greater symptom severity (14).

#### 2.6.2 Photo-stress recovery time (PSRT)

PSRT is the time (in seconds) taken for visual acuity to return to normal after the retina has been bleached by a bright light source. The test involves exposing the eye to the light from the ophthalmoscope for 30 s and measuring the time taken for acuity to return to within one line of pre-bleach acuity. The PSRT can be used to differentiate between retinal (macular) and post-retinal (e.g., optic nerve) diseases.

#### 2.6.3 Contrast sensitivity (CS)

Contrast sensitivity test measures a patient’s ability to differentiate between finer increments of light versus dark (contrast). CS was assessed using the Melbourne Edge Test. This test presents 20 circular patches containing edges with reducing contrast. Accurate identification of the orientation of the edges on the patches provides a measure of contrast sensitivity in decibel units, where dB = −10log10 contrast (15).

#### 2.6.4 Visual acuity test (VAT)

VAT measures the eye’s ability to see and read a letter or a symbol from a distance. During a VAT using a Snellen chart, random letters and numbers of varying sizes are displayed on a chart 6 meters away from the patient. The patient was required to cover one eye as they read the letters or numbers from top to bottom. Results are presented as a fraction ranging from 6/150 to 6/6 (reflecting normal vision) (16). Decimal notations can be calculated by dividing 6 meters by the participant’s corresponding score on the VAT. A mean score for tests conducted on the left and right eye (unaided) at each visit was calculated to evaluate changes over time.

#### 2.6.5 Tear film break-up time (TBUT)

TBUT is the time taken for the first dry spot to appear on the cornea after a full blink. TBUT is a method for assessing tear film stability and evaporative dry eye, and is a standard diagnostic procedure used in dry eye clinics. In TBUT test, sodium fluorescein dye is added to the eye, and the tear film is examined under the slit lamp while the patient avoids blinking until tiny dry spots develop. Generally, greater than 10 s is considered normal, 5 to 10 s marginal, and less than 5 s low. Short tear break-up time is a sign of a poor tear film, and the longer it takes, the more stable the tear film (17). A mean score for tests conducted on the left and right eye at each visit was calculated to evaluate changes over time.

### 2.7 Exploratory outcome measures

#### 2.7.1 PROMIS sleep disturbance and sleep-related impairment scale (PROMIS sleep)

The PROMIS Sleep is a validated - self-report questionnaire that assesses sleep quality and sleep-related impairment over the last 7 days. The measure comprises 16 items, creating 2 component scores (sleep disturbance and sleep-related impairment) (18).

#### 2.7.2 Everyday life attention scale (ELAS)

The ELAS was developed as a self-report questionnaire for the evaluation of attention in everyday life that takes into account different situational contexts. The ELAS contains questions about several attentional capacities in a variety of situations (reading a book, watching a movie, performing an indoor activity, attending a lecture/ open evening, having a conversation, doing an assignment/ administration, preparing a meal, cleaning up, driving a car) which are rated on a scale based on how long the respondent can engage in the task without a break, how well he/she can focus on the task, and his/her level of motivation to do the task well (19).

### 2.8 Safety outcome measures

The tolerability of capsule intake was assessed monthly through an online question about the experience of any adverse events. Researchers also asked about adverse events at visits 2 and 3, and participants were requested to contact researchers if they experienced any adverse reactions. Several safety blood measures were also collected comprising a full blood examination (hemoglobin, red blood cell count, hematocrit, mean corpuscular hemoglobin, mean corpuscular volume, mean corpuscular hemoglobin concentration, red blood cell distribution width, white cell count, neutrophils, lymphocytes, monocytes, eosinophils, basophils, platelets, and mean platelet volume), liver function test (aspartate transaminase, alanine transaminase, alkaline phosphatase, gamma-glutamyl transferase, bilirubin (total), total protein, globulin, and albumin), renal function test (urea, creatinine, estimated glomerular filtration rate, sodium, potassium, chloride, bicarbonate, and anion Gap), and blood lipid profile (cholesterol, triglycerides, high-density lipoprotein, and low-density lipoprotein).

### 2.9 Sample size calculations

An *a priori* power analysis was carried out to estimate the required sample size. In a study on the effects of LZ supplementation on high-screen users, effect sizes on various outcome measures associated with eye health ranged from 0.25 to 1.7 (9). As some ophthalmic outcome measures had moderate effect sizes of 0.6 to 0.8, an effect size of 0.7 was anticipated. Assuming a power of 80% and a type one error rate of 5%, the number of participants per group required to find an effect based on a single outcome measure was estimated as 26 (52 participants in total). Assuming a 20% dropout rate, it was planned to recruit 35 participants per group (70 participants in total), which was hypothesised to give enough power to find an effect compared to the placebo, even after dropouts.

### 2.10 Statistical analysis

For baseline data, an independent samples t-test was used to compare group data for continuous variables, and a Pearson’s Chi-square test was used to compare categorical data. Outcome analyses were conducted on the full analysis set (FAS), per protocol set (PPS), and safety analysis set (SAS), with all participants retained in originally allocated groups. FAS was defined as the subset of participants who were randomized and consumed at least one dose of the investigational product and who had available efficacy data. PPS was defined as the subset of randomized participants, who consumed at least one dose of the trial product, had available efficacy data, and had no major protocol deviations (e.g., withdrew from the study, consumed less than 80% of the investigational product, commenced prohibited concomitant medications, had missing data, and/or completed assessments outside proposed visit windows). Generalized Linear Mixed Models (GLMM) assessed differences between intervention groups on primary and secondary outcomes over time. Changes in scores from baseline (day 0) to day 180 were used to examine group differences with age, sex, BMI, and corresponding baseline scores included as covariates. To examine within-group changes over time, the GLMM was used where all data collection time points were included. The time points considered for each eye assessment were days 0, 90, and 180; and for self-report questionnaires, days 0 through 180. Random intercepts were utilized in each model, and covariates of age, sex, and BMI were included. Where appropriate, gamma (with log link function) and normal (with identity link function) target distributions were used. Applicable covariance structures were used to model correlation related with repeated time measurements in gamma models. All data were analyzed using SPSS (version 29; IBM, Armonk, NY), and the critical *p*-value was set at *p* ≤ 0.05 (two-sided for baseline data and one-sided for the analysis of outcome measures).

## 3 Results

### 3.1 Study population

A total of 153 people completed the online screening questionnaire, 88 people underwent a telephone screening, and 70 people attended an in-person screening assessment. Of the 88 people who participated in the telephone screening, the most common reasons for exclusion were failure to attend their in-person assessment appointment (*n* = 11) and withdrew consent (*n* = 4).

### 3.2 Baseline questionnaire and demographic information

Baseline demographic and clinical characteristics are detailed in Table 1. Analyses revealed that the groups were similarly matched with no statistically significant between-group differences, except for the physical activity category, where participants in the LZ group were less physically active (*p* = 0.003).

**Table 1:** Baseline sociodemographic and clinical characteristics.

	LZ (*n* = 35)	Placebo (*n* = 35)	*p*-value	
Age	Mean	48.26	46.07	0.449^a^
	SD	12.06	12.04	
	Min	24.80	20.50	
	Max	65.00	65.40	
Sex	Female (n)	16	16	1.00^b^
	Male (n)	19	19	
BMI	Mean	26.12	25.59	0.441^a^
	SD	2.66	3.03	
	Min	21.37	18.35	
	Max	29.93	29.91	
Systolic blood pressure (mmHg)	Mean	123.77	123.03	0.833^a^
	SD	14.99	14.41	
	Min	94.00	92.00	
	Max	158.00	148.00	
Diastolic blood pressure (mmHg)	Mean	77.69	79.11	0.571^a^
	SD	11.45	9.43	
	Min	59.00	61.00	
	Max	107.00	98.00	
Marital status	Single	15	14	0.808^b^
	Married/defacto	20	21	
Educational level	Secondary	20	14	0.348^b^
	Tertiary	8	12	
	Post-graduate	7	9	
International Physical Activity Questionnaires category	Low	24	10	0.003^b^
	Moderate	10	21	
	High	1	4	
Occupation	Retired	2	3	0.767^b^
	Professional	12	17	
	Services and sales worker	4	2	
	Unemployed	2	0	
	Technicians and associated trades	2	3	
	Elementary occupation	1	0	
	Student	2	3	
	Clerical support worker	3	2	
	Craft and related trades worker	1	1	
	Manager	6.00	4.00	
STT (mm)	Mean	21.20	20.83	0.896^b^
	SD	12.19	11.55	
	Min	0.00	0.00	
	Max	35.00	35.00	
PSRT (sec)	Mean	9.37	8.34	0.087^a^
	SD	14.71	9.51	
	Min	1.00	1.00	
	Max	84.00	45.00	
TBUT (sec)	Mean	29.30	27.51	0.949^a^
	SD	22.29	20.31	
	Min	8.50	7.00	
	Max	104.50	95.00	
CS (decibel units)	Mean	20.77	21.23	0.786^a^
	SD	1.99	1.54	
	Min	16.00	19.00	
	Max	24.00	24.00	
VA (meter)	Mean	12.28	14.41	0.549^a^
	SD	13.07	16.31	
	Min	6.0	6.0	
	Max	60.00	90.0	
VFS total score	Mean	11.77	10.40	0.552^a^
	SD	11.29	7.55	
	Min	0.00	3.00	
	Max	41.00	32.00	
CVS-Q total score	Mean	8.40	6.06	0.145^a^
	SD	8.32	4.30	
	Min	0.00	1.00	
	Max	43.00	19.00	
PROMIS sleep disturbance (T-score)	Mean	50.41	50.68	0.877^a^
	SD	8.07	6.47	
	Min	28.90	35.50	
	Max	66.00	66.50	
PROMIS sleep-related impairment (T-score)	Mean	51.01	50.73	0.891^a^
	SD	9.73	7.59	
	Min	30.00	33.70	
	Max	70.60	70.70	
ELAS total score	Mean	102.83	95.28	0.244^a^
	SD	29.26	24.31	
	Min	25.02	39.04	
	Max	159.70	145.17	

a Independent-Samples *T*-Test (two-sided).

b Pearson Chi-Square Test.

### 3.3 Outcome measures

#### 3.3.1 Primary outcome measures

##### 3.3.1.1 STT

**Figure 2:**
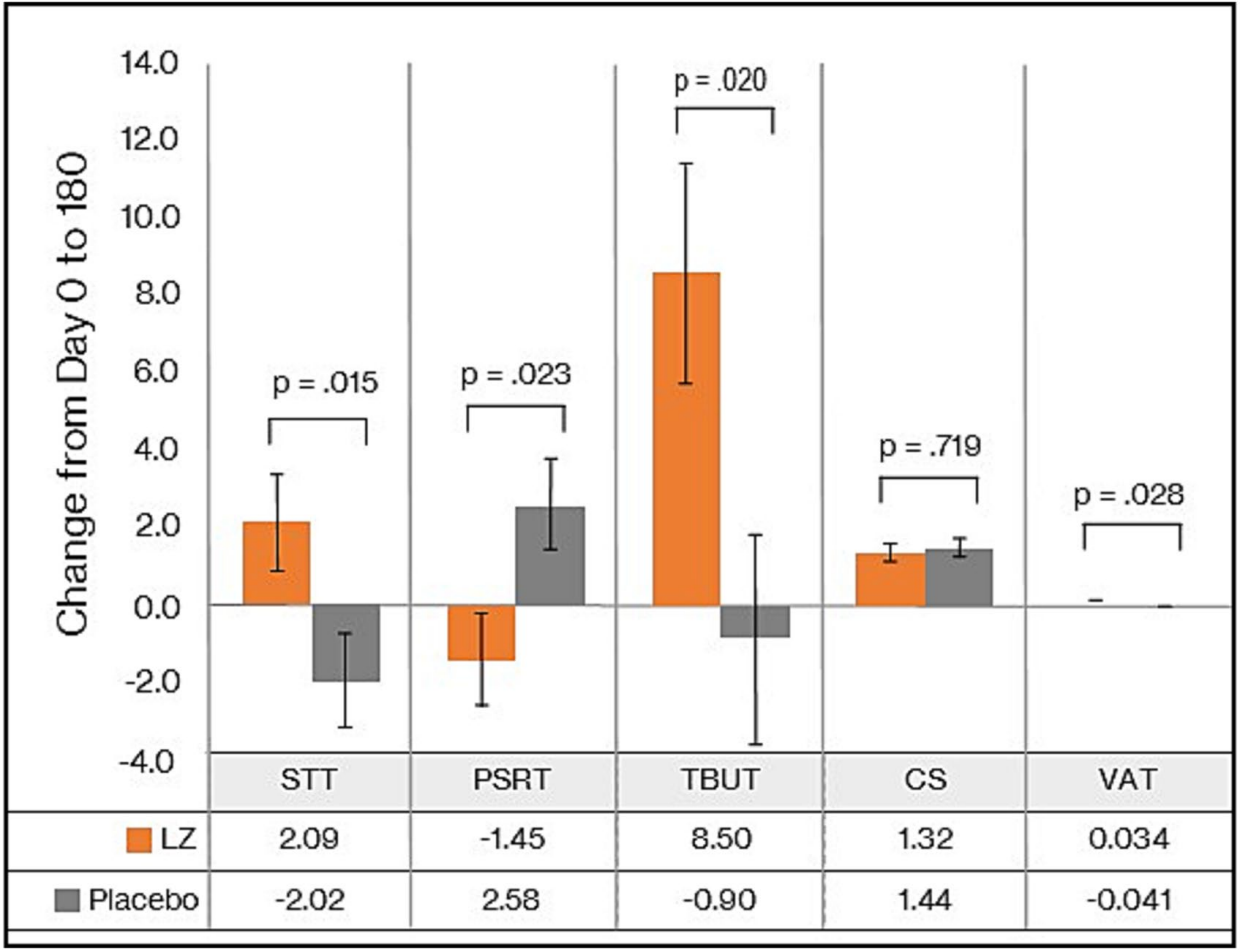
Change in eye assessments from day 0 to 180 (FAS).

As demonstrated in Table 2, the GLMM revealed a statistically significant difference in change in STT scores from day 0 to day 180 (*p* = 0.015). As detailed in Table 2 and Figure 2, from baseline to day 180, there was a non-significant increase of 2.09 mm in the LZ group (*p* = 0.123) and a non-significant 2.02 mm decrease in the placebo group (*p* = 0.136) [Cohen’s D effect size (ES) = 0.68]. An analysis of the PPS revealed similar findings (see Supplementary Table S1), as demonstrated by statistically significant group differences (*p* = 0.025) and an ES of 0.69.

**Table 2:** Eye assessment results completed at each visit (estimated marginal means) (FAS).

			Day 0	Day 90	Day 180	Change from baseline^a^	*p*-value^b^	*p*-value^a^	Cohen’s D effect size^a^
STT (mm)	Placebo (*n* = 31)	Mean	21.19	20.94	19.38	−2.02	0.136	0.015	0.68
		SE	1.88	1.90	1.76	1.14			
	LZ (*n* = 29)	Mean	20.66	19.70	22.74	2.09	0.123		
		SE	1.84	1.79	2.09	1.18			
PSRT (sec)	Placebo (*n* = 31)	Mean	9.10	11.49	11.36	2.58	0.116	0.023	0.64
		SE	1.11	1.47	1.45	1.19			
	LZ (*n* = 29)	Mean	9.72	8.62	7.91	−1.45	0.143		
		SE	1.19	1.10	1.04	1.23			
TBUT (sec)	Placebo (*n* = 31)	Mean	28.21	30.57	27.30	−0.90	0.694	0.020	0.65
		SE	2.96	3.31	2.95	2.72			
	LZ (*n* = 29)	Mean	25.51	28.53	33.00	8.50	0.005		
		SE	2.69	3.09	3.62	2.81			
Contrast sensitivity (db)	Placebo (*n* = 31)	Mean	21.19	21.75	22.34	1.44	0.001	0.719	0.10
		SE	0.24	0.27	0.27	0.22			
	LZ (*n* = 29)	Mean	20.79	21.66	22.30	1.32	< 0.001		
		SE	0.24	0.27	0.28	0.23			
Visual acuity (VAT)	Placebo (*n* = 31)	Mean	0.664	0.646	0.627	−0.041	0.148	0.028	0.62
		SE	0.052	0.052	0.050	0.023			
	LZ (*n* = 29)	Mean	0.768	0.740	0.782	0.034	0.646		
		SE	0.061	0.059	0.063	0.024			

a Estimated marginal means, *p*-values, and Cohen’s D Effect sizes were calculated based on changes in scores from day 0 to day 180 using GLMM adjusted for age, sex, BMI, and corresponding baseline scores.

b *P*-values are generated from repeated measures GLMM adjusted for age, sex, and BMI (time x group interaction).

##### 3.3.1.2 VFS

As demonstrated in Table 3, based on the GLMM, there was no statistically significant difference in change in VFS scores from day 0 to 180 (*p* = 0.202). In the LZ and placebo groups, there were statistically significant within-group changes over time in VFS scores (*p* < 0.001). PPS data revealed similar non-significant group differences in changes in VFS scores (see Supplementary Table S2).

**Table 3:** Self-report questionnaires completed at each time point (estimated marginal means) (FAS).

			Day 0	Day 30	Day 60	Day 90	Day 120	Day 150	Day 180	Change from baseline^a^	*p*-value^b^	*p*-value^a^
VFS	Placebo (*n* = 31)	Mean	10.38	6.98	6.21	6.44	4.73	4.31	4.76	−6.55	< 0.001	0.202
		SE	1.43	1.43	1.45	1.45	1.45	1.45	1.45	1.26		
	LZ (*n* = 29)	Mean	11.54	11.17	8.38	9.25	8.04	7.76	7.43	−4.20	< 0.001	
		SE	1.43	1.44	1.44	1.46	1.47	1.47	1.47	1.30		
CVS-Q	Placebo (*n* = 31)	Mean	6.06	5.40	4.72	4.81	3.98	4.01	3.98	−2.78	0.005	0.787
		SE	1.02	1.02	1.04	1.04	1.04	1.04	1.04	0.85		
	LZ (*n* = 29)	Mean	8.17	8.36	6.27	6.03	5.43	4.99	5.49	−2.45	< 0.001	
		SE	1.02	1.04	1.04	1.04	1.04	1.04	1.06	0.88		
ELAS	Placebo (*n* = 31)	Mean	95.15	–	96.23	–	100.75	–	96.84	1.08	0.610	0.469
		SE	4.31	–	4.47	–	4.71	–	4.50	3.67		
	LZ (*n* = 29)	Mean	102.58	–	96.59	–	102.91	–	106.48	4.95	0.292	
		SE	4.65	–	4.44	–	4.85	–	5.01	3.79		
PROMIS Sleep Disturbance	Placebo (*n* = 31)	Mean	50.75	48.86	48.95	47.40	49.00	48.32	47.25	−4.43	0.001	0.893
		SE	1.46	1.41	1.44	1.40	1.44	1.42	1.39	2.07		
	LZ (*n* = 29)	Mean	50.43	49.71	49.64	48.23	50.01	49.69	48.32	−4.02	0.060	
		SE	1.46	1.45	1.45	1.43	1.50	1.49	1.45	2.14		
PROMIS Sleep-Related Impairment	Placebo (*n* = 31)	Mean	50.59	48.28	47.76	46.46	48.73	48.11	47.13	−3.86	0.005	0.919
		SE	1.62	1.54	1.56	1.52	1.60	1.58	1.54	1.56		
	LZ (*n* = 29)	Mean	50.98	51.18	49.08	47.98	49.32	48.70	47.19	−4.09	0.003	
		SE	1.64	1.66	1.60	1.59	1.64	1.62	1.57	1.62		

a Estimated marginal means, *p*-values, and Cohen’s D Effect sizes were calculated based on changes in scores from day 0 to day 180 using GLMM adjusted for age, sex, BMI, and corresponding baseline scores.

b *p*-values are generated from repeated measures GLMM adjusted for age, sex, and BMI (time x group interaction).

#### 3.3.2 Secondary outcome measures

##### 3.3.2.1 TBUT

As demonstrated in Table 2, the GLMM revealed a statistically significant difference in change in TBUT scores from day 0 to 180 (*p* = 0.020). As detailed in Table 2 and Figure 2, from baseline to day 180, there was a statistically significant increase of 8.50 s in the LZ group and a non-significant 0.90 s decrease in the placebo group (ES = 0.65). An analysis of the PPS revealed similar findings (see Supplementary Table S1), as demonstrated by statistically significant group differences (*p* = 0.045) and an ES of 0.62.

##### 3.3.2.2 PSRT

As demonstrated in Table 2, based on the GLMM, there was a statistically significant difference in change in PSRT scores from day 0 to 180 (p = 0.045). As detailed in Table 2 and Figure 2, from baseline to day 180, there was a non-significant decrease of 1.45 s in the LZ group (*p* = 0.143) and a non-significant 2.58 s increase in the placebo group (*p* = 0.116) (ES = 0.64). An analysis of the PPS revealed similar findings (see Supplementary Table S1), as demonstrated by an ES of 0.46, although the group difference was no longer statistically significant (*p* = 0.132).

##### 3.3.2.3 CS

As demonstrated in Table 2, based on the GLMM, there was no statistically significant difference in change in CS sores from day 0 to 180 (*p* = 0.719). PPS data revealed similar non-significant group differences in changes in CS scores (see Supplementary Tables S1, S2).

##### 3.3.2.4 VAT

As demonstrated in Table 2, the GLMM revealed a statistically significant difference in change in VAT scores from day 0 to 180 (*p* = 0.028). As detailed in Table 2 and Figure 2, from baseline to day 180, there was a non-significant increase of 0.03 in VAT scores in the LZ group (*p* = 0.646) and a non-significant decrease of 0.041 in the placebo group (*p* = 0.148) (ES = 0.62). An analysis of the PPS revealed similar findings (see Supplementary Table S1), as demonstrated by an ES of 0.59, although the group difference was no longer statistically significant (*p* = 0.053).

##### 3.3.2.5 CVS-Q

As demonstrated in Table 3, based on the GLMM, there was no statistically significant difference in change in CVS-Q scores from day 0 to 180 (*p* = 0.787). In the LZ and placebo groups, there were statistically significant within-group changes over time in VFS scores (*p* < 0.001 and *p* = 0.005, respectively). PPS data revealed similar non-significant group differences in changes in CVS-Q scores (see Supplementary Table S2).

#### 3.3.3 Exploratory outcome measures

##### 3.3.3.1 PROMIS sleep

As demonstrated in Table 4, based on the GLMM, there was no statistically significant difference in change in PROMIS Sleep Disturbance (*p* = 0.893) and Sleep-Related Impairment (*p* = 0.919) scores from day 0 to 180. PPS data revealed similar non-significant group differences in changes in PROMIS Sleep scores (see Supplementary Table S2).

**Table 4:** Possibly or probably related AEs by class and term.

AE Class	Diagnosis or symptom	Placebo (*N* = 35)	LZ (*N* = 35)
Muscular	n	1 (2.9%)	0 (0%)
	Sore joints	1 (2.9%)	0 (0%)
Gastrointestinal	n	1 (2.9%)	1 (2.9%)
	Stomach pain/ bloating	1 (2.9%)	1 (2.9%)
Sleep	n	2 (5.7%)	0 (0%)
	Worsened sleep	2 (5.7%)	0 (0%)
Dermatological	n	2 (5.7%)	0 (0%)
	Swollen lips	1 (2.9%)	0 (0%)
	Face rash	1 (2.9%)	0 (0%)
Ocular	n	1 (2.9%)	2 (5.7%)
	Sore eyes/ eye irritation	1 (2.9%)	2 (5.7%)
All Adverse events	n	7 (20.0%)	3 (8.6%)
Number of participants experiencing treatment-related AE*	n	5 (14.3%)	3 (8.6%)

^*^Some participants experienced more than one treatment-related AE.

##### 3.3.3.2 ELAS

As demonstrated in Table 3, based on the GLMM, there was no statistically significant difference in change in ELAS scores from day 0 to 180 (*p* = 0.469). In the LZ and placebo groups, there were no statistically significant within-group changes over time in ELAS scores (*p* = 0.292 and *p* = 0.610, respectively). PPS data revealed similar non-significant group differences in changes in ELAS scores (see Supplementary Table S3).

### 3.4 Intake of supplements

IP bottles with remaining capsules were returned on visits 2 and 3. Based on these details, 98% of participants who completed the study took over 80% of their capsules.

### 3.5 Efficacy of participant blinding

To assess the effectiveness of condition concealment during the trial, participants predicted their condition allocation (i.e., placebo, LZ, or unsure) at the end of the study. Overall group concealment was high, as 66% of participants in the placebo group and 68% of participants in the LZ group were unsure or incorrectly guessed treatment allocation.

### 3.6 Adverse reactions and treatment discontinuation

Participants reported no serious adverse events, and there was a tendency for a greater frequency of adverse events in the placebo group. Table 4 details the adverse events classified as possibly or probably related to the investigational products. In the placebo group, 14.3% of participants experienced a treatment-related adverse event, and in the LZ group, 8.6% of participants experienced a treatment-related adverse event.

No participants experienced clinically significant changes in blood markers over time (complete blood count, liver function test, blood lipids and renal function test), with concentrations remaining within or close to established reference ranges and none reaching clinically significant levels. Based on the FAS, there were statistically significant group differences in changes in concentrations of neutrophils (*p* = 0.046), albumin (*p* = 0.049), anion gap (*p* = 0.045), and estimated glomerular filtration rate (*p* = 0.024). However, these differences between groups must be viewed tentatively as the number of analyses conducted increases the likelihood of type 1 error. Moreover, changes in these markers were small and not clinically meaningful. There were also no group differences in weight, BMI, blood pressure, or pulse rate changes over the 6-month study period.

A total of 10 people discontinued the study. Six people in the LZ group withdrew from the study, three due to adverse effects believed to be associated with capsule intake (mild severity), one due to personal stressors, one due to inconsistent capsule intake, and one where no reason was given. Reasons for study withdrawal in the three people in the LZ group comprised worsening eye symptoms (*n* = 1), stomach bloating (*n* = 1), and pain above the right eye (*n* = 1). In the placebo group, four people withdrew from the study, two due to adverse events believed to be associated with capsule intake (mild severity), one due to unexpected travel, and one where no reason was given. Reasons for study withdrawal in the two people in the placebo group comprised swollen lips (*n* = 1) and stomach pain/ bloating (*n* = 1).

## 4 Discussion

In this randomized, double-blind, placebo-controlled trial, the effects of 6 months of LZ supplementation (Lute-gen^®^) on visual fatigue, CVS, dry eyes, visual performance, sleep quality, and attention were examined in high users of electronic screens. Outcome measures comprised a combination of self-report questionnaires and ophthalmic examinations, with the VFS, a self-report measure of visual fatigue, and the STT, a measure of dry eyes/ tear production, comprising the primary outcome measures. Compared to the placebo, LZ supplementation did not have a differential effect on the VFS score but there was a statistically significant group difference in changes in STT. Moreover, LZ supplementation was associated with group differences in changes in several other ophthalmic examinations, including the TBUT, PSRT, and VAT. However, it is important to note that the group difference in STT and PSRT changes over time were the result of a combination of improvements, albeit non-significant, in the LZ group, and a worsening, albeit non-significant, in the placebo group. As the observed group difference in VAT was largely due to a non-significant trend of decreased performance in the placebo group, this finding should be considered cautiously. Despite the positive changes in eye health and function based on ophthalmic examinations, improvements from LZ supplementation were not supported by group differences in changes in self-report measures. This suggests that even though measures of eye health and dry eyes through more objective ophthalmic examinations occurred, these changes were not necessarily of enough significance for participants to realize symptomatic subjective changes.

Dry eye disease is a multifactorial disease characterized by discomfort, visual disturbance, and tear film instability with potential damage to the ocular surface. Estimates of the prevalence of dry eye disease vary based on the population examined, the definition used, and the assessment methods utilized, with global prevalence rates ranging from 5 to 34% (20). Risk factors for dry eyes include ageing, female gender, contact lens use, history of ocular or laser refractive surgery, and systemic diseases such as Sjogren’s syndrome, rheumatoid arthritis, gout, thyroid disease, and autoimmune disorders. In addition, environmental factors such as low room humidity, high temperature, air pollution, and certain lighting conditions can exacerbate dry eyes. Moreover, excessive digital screen use can contribute to dry eyes (20). A commonly-accepted hypothesis for the relationship between digital screen use and dry eye disease is that digital screen use alters blinking dynamics, leading to ocular dryness (2). Through overexposure to blue light, electronic screen use can also contribute to the excessive production and accumulation of free oxygen radicals in mitochondria and photosensitive molecules (3, 4).

Based on the results from two ophthalmic examinations comprising the STT and TBUT, this study demonstrated that LZ supplementation for 6 months can increase tear production. The STT is a commonly used measure of total tear secretion (21), and the TBUT provides a measure of tear film instability (22, 23). LZ are the main carotenoids in the human macula, often referred to as macular pigments. Even though further investigation is required to understand the photo-protective actions associated with LZ supplementation, it may be via their antioxidant and anti-inflammatory actions. Several studies have demonstrated that lutein inhibits the pro-inflammatory cytokine cascade and the transcription factor, nuclear factor-kB. There is also evidence that LZ reduce reactive oxygen species production and the expression of inducible nitric oxide synthase (2, 5). Another protective effect of lutein may be through its ability to filter blue light, thereby reducing phototoxic damage to photoreceptor cells (11). In a study on adults with high screen use, LZ supplementation for 6 months was associated with improvements in macular pigment optical density (MPOD) and several visual performance measures (9). In addition to changes in tear production, an improvement in PSRT was also demonstrated in this study. PSRT is an objective quantitative measure of macular function, and several diseases influencing central vision, including age-related macular degeneration, central serous retinopathy, retinal detachments, and retinitis pigmentosa, can affect recovery time (24, 25). In meta-analyses examining the association between LZ supplementation, MPOD, and visual function, it was concluded that LZ supplementation increased MPOD, and LZ intake/ supplementation and MPOD are associated with reduced photostress recovery and improved visual acuity (26–29). Therefore, the positive changes in PSRT identified in this study are consistent with previous trials conducted in the area. However, despite several improvements in ophthalmic examinations being identified, this did not translate into between-group differences in changes in self-report questionnaires assessing dry eyes, visual fatigue, eye soreness, or other eye-related symptoms associated with high electronic screen use. There are several reasons for these inconsistent findings. It may be that even though tear production increased, the changes were of insufficient intensity to result in meaningful and clinically noticeable symptomatic improvements. However, it is important to note that placebo responses are common in clinical trials, with subjective evaluations particularly susceptible to placebo responses (30). Such placebo responses did not occur when investigator-administered ophthalmic examinations were undertaken. Another consideration is that baseline scores on the self-report questionnaires were low, indicating that despite recruiting high electronic screen users, complaints associated with dry eyes and computer vision syndrome were not highly prevalent in the recruited population. This suggests that the recruited population did not experience dry eye and visual symptoms of sufficient severity to result in noticeable problems for participants, and/or visual-related symptoms were accepted as part of participants’ everyday experiences. Even though there is no consensus on the diagnostic criteria of dry eye in STT, a reading of less than 5 mm indicates dry eyes and less than 10 mm marginally dry eyes (21). At baseline, the mean STT score of participants in this study was approximately 20 mm, indicating normal tear production in most participants. Moreover, despite research demonstrating excessive electronic screen time can contribute to sleep and attention problems (31, 32), such difficulties were not present in the population examined.

### 4.1 Limitations and directions for future research

Although the ophthalmic examinations completed in the study are considered acceptable measures, they have several limitations. For example, the TBUT use of fluorescein dye does not allow for observation of the physiological state of the ocular surface. Moreover, the breakup time depends on the amount of fluorescein dye used, and it is sometimes difficult to determine when the tear film begins to breakup. This can affect the reproducibility of results (22, 23). In several studies, the STT did not reliably detect the efficacy of drugs in patients undergoing treatment for dry eye, and its weaknesses included poor repeatability, low sensitivity and specificity, and sharp patient discomfort. Changes in light, room humidity, temperature and patient’s anxiety can also influence the reproducibility of results (21). Therefore, additional examinations will be important to validate the results of this trial. This is particularly important due to the inconsistent findings in this study where positive changes in ophthalmic examinations were not supported by changes in subjective outcome measures. Some non-invasive measures of dry eye include thermography, anterior segment optical coherence tomography, meibography, and interferometry (21). MPOD assessments to examine the effects of LZ supplementation on MPOD and visual changes would also be useful. In a study by Stringham and colleagues (9), LZ supplementation improved MPOD, and MPOD was correlated with improvements in visual performance. Assessing for, and controlling for, changes in diet quality and the intake of carotenoid-rich foods will be helpful to ensure visual changes are not the result of changes in dietary patterns during the study. However, it would be expected that if there were any changes in the dietary intake of LZ and other carotenoids during the study, it would be similar across the two groups. The recruitment of participants with identified dry eye syndrome, CVS, poor sleep and attentional problems will also help to understand the effects of LZ supplementation in people presenting with such difficulties. In this study, high users of electronic screens were recruited as this is associated with an increased risk of dry eye symptoms and visual fatigue. However, as was previously discussed, many recruited participants did not exhibit such problematic symptoms. Moreover, the exploratory outcomes comprising sleep and attention require further investigation in populations experiencing problems in these areas. Such factors require further consideration when developing eligibility and recruitment strategies in the future. Finally, sunflower oil was used as the placebo/ control condition, and it could be argued that this may have beneficial effects on eye health. However, LZ soft gels also contained similar concentrations of sunflower oil as a carrier. Therefore, the additional effects of LZ on eye health could still be elucidated. Moreover, daily concentrations of sunflower oil were small and delivered at levels significantly lower than those used in interventional studies (33).

In summary, the results from this study provide some support for the beneficial effects of 6 months of LZ supplementation on regular users of electronic screens. Future investigations to expand on the current findings will be important in specifically targeted populations experiencing dry eyes and visual fatigue and utilizing a range of validated objective and subjective measures to examine changes in eye health and visual symptoms over time. Moreover, to better understand the effects of LZ on sleep and attention, more research is required on individuals experiencing difficulties in these areas utilizing validated objective and subjective measures.

## Data Availability

The raw data supporting the conclusions of this article will be made available by the authors, without undue reservation.
