# Supplementary material for: The effects of lutein/ zeaxanthin (Lute-gen®) on eye health, eye strain, sleep quality, and attention in high electronic screen users: a randomized, double-blind, placebo-controlled study
Source: Front Nutr. 2025 Feb 3;12:1522302. doi: 10.3389/fnut.2025.1522302 (PMC11830589; doi:10.3389/fnut.2025.1522302)
Supplement: Supplementary file 1 [file Table_1.docx]

Supplementary Material

The effects of lutein/ zeaxanthin (Lute-gen^®^) on eye health, eye strain, sleep quality, and attention in high electronic screen users: a randomized, double-blind, placebo-controlled study

Lopresti, A.L.^1,2*^, Smith, S.J.S.^1^

^1^Clinical Research Australia, Perth, Western Australia, 6023, Australia

^2^Healthy Ageing Research Centre and Discipline of Psychology, College of Science, Health, Engineering and Education, Murdoch University, Perth, Western Australia, 6150, Australia

*** Correspondence:** Corresponding Author: adrian@clinicalresearch.com.au

**Table 1. Eye assessment results completed at each visit (estimated marginal means) (PPS)**

|  |  |  | Day 0 | Day 90 | Day 180 | p-value^a^ | p-value^b^ |
| --- | --- | --- | --- | --- | --- | --- | --- |
| STT (mm) | Placebo (n = 26) | Mean | 20.45 | 21.21 | 19.21 | 0.333 | 0.046 |
|  |  | SE | 2.20 | 2.28 | 2.06 |  |  |
|  | LZ (n = 25) | Mean | 19.97 | 19.80 | 22.31 | 0.098 |  |
|  |  | SE | 2.19 | 2.18 | 2.45 |  |  |
| PSRT (sec) | Placebo (n = 26) | Mean | 8.63 | 10.83 | 11.34 | 0.081 | 0.105 |
|  |  | SE | 1.21 | 1.52 | 1.59 |  |  |
|  | LZ (n = 25) | Mean | 9.29 | 7.63 | 8.39 | 0.514 |  |
|  |  | SE | 1.33 | 1.11 | 1.20 |  |  |
| TBUT (sec) | Placebo (n = 26) | Mean | 28.81 | 32.72 | 27.41 | 0.573 | 0.016 |
|  |  | SE | 3.28 | 3.72 | 3.12 |  |  |
|  | LZ (n = 25) | Mean | 23.65 | 27.35 | 31.22 | 0.004 |  |
|  |  | SE | 2.74 | 3.20 | 3.62 |  |  |
| Contrast Sensitivity (db) | Placebo (n = 26) | Mean | 21.12 | 21.77 | 22.44 | 0.001 | 0.995 |
|  |  | SE | 0.28 | 0.29 | 0.30 |  |  |
|  | LZ (n = 25) | Mean | 20.98 | 21.58 | 22.23 | 0.001 |  |
|  |  | SE | 0.29 | 0.30 | 0.30 |  |  |
| Visual acuity | Placebo (n = 26) | Mean | 0.654 | 0.645 | 0.617 | 0.172 | 0.157 |
|  |  | SE | 0.063 | 0.062 | 0.059 |  |  |
|  | LZ (n = 25) | Mean | 0.719 | 0.723 | 0.756 | 0.244 |  |
|  |  | SE | 0.070 | 0.071 | 0.074 |  |  |

Results (estimated means) are generated from generalised mixed-effects models adjusted for age, sex, and BMI. ^a^P-values are generated from repeated measures generalised mixed-effects models adjusted for age, sex, and BMI (time effects baseline and day 180). ^b^P-values are generated from repeated measures generalised mixed-effects models adjusted for age, sex, and BMI (time x group interaction).

**Table 2. Mean change in eye assessments from day 0 to 180 (estimated marginal means) (PPS)**

|  | Group | N | Mean change  (day 0 to 180) | SE | 95% Confidence Interval | | Cohen’s D Effect Size | P-value |
| --- | --- | --- | --- | --- | --- | --- | --- | --- |
|  |  |  |  |  | Lower | Upper |  |  |
| STT (mm) | LZ | 25 | 2.34 | 1.27 | -0.22 | 4.91 | 0.66 | 0.025 |
|  | Placebo | 26 | -1.79 | 1.25 | -4.30 | 0.72 |  |  |
| PSRT (sec) | LZ | 25 | -0.52 | 1.35 | -3.24 | 2.21 | 0.44 | 0.132 |
|  | Placebo | 26 | 2.39 | 1.32 | -0.28 | 5.06 |  |  |
| TBUT (sec) | LZ | 25 | 7.94 | 3.00 | 1.90 | 13.99 | 0.59 | 0.045 |
|  | Placebo | 26 | -0.76 | 2.95 | -6.70 | 5.17 |  |  |
| Contrast Sensitivity (db) | LZ | 25 | 1.17 | 0.22 | 0.71 | 1.62 | 0.21 | 0.465 |
|  | Placebo | 26 | 1.40 | 0.22 | 0.95 | 1.84 |  |  |
| Visual acuity | LZ | 29 | 0.044 | 0.027 | -0.010 | 0.097 | 0.57 | 0.053 |
|  | Placebo | 31 | -0.031 | 0.026 | -0.084 | 0.022 |  |  |

Results (estimated means) are generated from generalised mixed-effects models adjusted for age, sex, BMI, and corresponding baseline values. P-values are generated from generalised mixed-effects models (change from day 0 to 180) adjusted for age, sex, BMI, and corresponding baseline valuess

**Table 3. Self-report questionnaires completed at each time point (estimated marginal means) (PPS)**

|  |  |  | Day 0 | Day 30 | Day 60 | Day 90 | Day 120 | Day 150 | Day 180 | p-value^a^ | p-value^b^ |
| --- | --- | --- | --- | --- | --- | --- | --- | --- | --- | --- | --- |
| VFS | Placebo (n = 26) | Mean | 10.37 | 6.56 | 5.66 | 6.25 | 4.41 | 4.18 | 4.91 | < .001 | 0.729 |
|  |  | SE | 1.60 | 1.60 | 1.60 | 1.60 | 1.60 | 1.60 | 1.60 |  |  |
|  | LZ (n = 25) | Mean | 12.83 | 11.27 | 8.87 | 9.62 | 8.27 | 8.15 | 7.67 | < .001 |  |
|  |  | SE | 1.63 | 1.63 | 1.63 | 1.64 | 1.64 | 1.63 | 1.63 |  |  |
| CVS-Q | Placebo (n = 26) | Mean | 6.04 | 5.35 | 4.88 | 4.69 | 3.96 | 4.04 | 4.00 | 0.007 | 0.798 |
|  |  | SE | 1.00 | 1.00 | 1.00 | 1.00 | 1.00 | 1.00 | 1.00 |  |  |
|  | LZ (n = 25) | Mean | 8.33 | 7.81 | 5.85 | 6.17 | 5.73 | 5.29 | 5.57 | < .001 |  |
|  |  | SE | 1.02 | 1.02 | 1.02 | 1.02 | 1.02 | 1.02 | 1.02 |  |  |
| ELAS | Placebo (n = 26) | Mean | 101.49 | - | 101.81 | - | 106.07 | - | 101.92 | 0.910 | 0.503 |
|  |  | SE | 5.15 | - | 5.20 | - | 5.42 | - | 5.17 |  |  |
|  | LZ (n = 25) | Mean | 98.34 | - | 98.39 | - | 101.94 | - | 105.23 | 0.080 |  |
|  |  | SE | 5.09 | - | 5.09 | - | 5.32 | - | 5.45 |  |  |
| PROMIS Sleep Disturbance | Placebo (n = 26) | Mean | 50.55 | 49.09 | 49.21 | 47.25 | 48.68 | 48.62 | 47.49 | 0.012 | 0.990 |
|  |  | SE | 1.74 | 1.69 | 1.70 | 1.62 | 1.67 | 1.67 | 1.63 |  |  |
|  | LZ (n = 25) | Mean | 50.81 | 49.42 | 48.27 | 47.57 | 48.97 | 48.97 | 47.57 | 0.010 |  |
|  |  | SE | 1.78 | 1.73 | 1.69 | 1.68 | 1.73 | 1.72 | 1.67 |  |  |
| PROMIS Sleep-Related Impairment | Placebo (n = 26) | Mean | 49.64 | 48.08 | 47.83 | 45.38 | 47.46 | 47.87 | 47.08 | 0.061 | 0.537 |
|  |  | SE | 1.83 | 1.77 | 1.77 | 1.67 | 1.75 | 1.76 | 1.73 |  |  |
|  | LZ (n = 25) | Mean | 51.14 | 51.52 | 48.25 | 47.54 | 49.16 | 48.45 | 46.66 | 0.002 |  |
|  |  | SE | 1.92 | 1.93 | 1.81 | 1.80 | 1.86 | 1.82 | 1.75 |  |  |

Results (estimated means) are generated from generalised mixed-effects models adjusted for age, sex, and BMI. ^a^P-values are generated from repeated measures generalised mixed-effects models adjusted for age, sex, and BMI (time effects baseline and day 180). ^b^P-values are generated from repeated measures generalised mixed-effects models adjusted for age, sex, and BMI (time x group interaction).
